# On the coupling and decoupling of mind wandering and perception: a shared metabolism account

**DOI:** 10.1093/texcom/tgad021

**Published:** 2023-11-21

**Authors:** M Bruckmaier, V Albrecht, I Tachtsidis, N Lavie

**Affiliations:** Institute of Cognitive Neuroscience, University College London, Alexandra House, 17-19 Queens Square, London WC1N 3AZ, United Kingdom; Institute of Cognitive Neuroscience, University College London, Alexandra House, 17-19 Queens Square, London WC1N 3AZ, United Kingdom; Department of Medical Physics and Biomedical Engineering, University College London, Malet Place Engineering Building, London WC1E 7JE, United Kingdom; Institute of Cognitive Neuroscience, University College London, Alexandra House, 17-19 Queens Square, London WC1N 3AZ, United Kingdom

**Keywords:** attention, cerebral metabolism, mind wandering, perceptual decoupling, perceptual load

## Abstract

**Introduction:**

Mind wandering (MW) has been associated with reduced responsiveness to external stimuli (“perceptual decoupling”). Conversely, increased perceptual demands of a task result in reduced MW. Here we propose a neurobiological account attributing the mutually-limiting relationship of MW and perception to brain-wide limits on cerebral metabolism. Since overall cerebral metabolism is known to remain constant, despite increased mental task demands, we tested whether increased perceptual processing load in a visual task will result in reduced oxygen metabolism in MW-related medial prefrontal cortex (mPFC) regions.

**Methods:**

We used broadband near-infrared spectroscopy to measure oxidation states of the cytochrome-c-oxidase enzyme (oxCCO), an intracellular marker of metabolism, in mPFC while sampling participants’ MW experiences during their performance of a visual task of either low (feature search) or high(conjunction search) perceptual load.

**Results:**

Increased perceptual load in the task resulted in reduced oxCCO signal in mPFC regions related to MW reports. High perceptual load was also found to specifically suppress detailed (and hence more metabolism-demanding) rather than vague MW.

**Discussion:**

Overall, the results support a shared metabolism account of the relationship between MW and perception and demonstrate that attentional-regulation of metabolism only supports ongoing detailed MW when perceptual processing demands are low.

## Introduction

Mind wandering (MW) is a ubiquitous experience, which can be so all-consuming that it may even override our experience of the external world. At times, we may find ourselves staring at the computer screen, but eventually realize that we did not perceive its content while our mind was elsewhere. Indeed, research has shown that MW is associated with reduced neural responses related to stimulus perception (Schooler et al. 2011; Smallwood and Schooler 2015). These include reports of reduced evoked potentials related to various visual and auditory stimuli during periods of MW (Braboszcz and Delorme 2011; Kam et al. 2011; Smallwood et al. 2012; Baird et al. 2014; Denkova et al. 2018), as well as reduced Blood-Oxygen-Level-Dependent (BOLD) response in visual cortex regions related to perception in studies when attention is directed towards internal tasks mimicking MW (e.g. mental imagery, autobiographical recall, Cohen et al. 2022; Zhang et al. 2022). These findings have been interpreted to suggest that MW can lead to a state of “perceptual decoupling”, caused by a disengagement of attention from processing stimuli in the external world, which in turn results in reduced brain responses related to perceptual processing (Smallwood et al. 2012; Franklin et al. 2013). Here we aim to clarify the cause for perceptual decoupling by relating this phenomenon to the Load Theory of attention, which accounts for selective attention effects within a limited capacity model (Lavie 2005; Lavie et al. 2014), together with recent work attributing capacity limits to brain-wide limits on neural oxygen metabolism.

Research into Load Theory has demonstrated that increased load on perceptual processing is not only associated with increased neural responses to task-related processing, but also with reduced distractor processing and the associated neural responses (Rees et al. 1997; Torralbo et al. 2016; Bruckmaier et al. 2020), as well as reduced occurrence of MW (Forster and Lavie 2009). Tasks of low perceptual load (e.g. visual search tasks involving feature-based search for angular letters, e.g. X/N, among Os), were found to involve not only higher levels of distractor processing, but also more frequent MW reports than tasks of high perceptual load (e.g. X/N search among similarly angular letters; Forster and Lavie 2009). Integrating this research body with the demonstrations of perceptual decoupling during MW, we propose that there is a bi-directional trade-off relationship between neural activity related to MW versus perception: Increased demands on neural activity related to perceptual processing in an attended task are associated with reduced neural activity related to any unattended processing, including MW, and conversely, periods of MW result in disengagement of attention from perceptual processing and thus result in reduced neural activity related to perception.

Importantly, to elucidate the underlying neural mechanisms for this bi-directional resource trade-off relationship between MW and perception, we derived insight from a neurobiological body of research, as follows. It is well established that neural firing is energetically expensive (Attwell and Laughlin 2001; Lennie 2003): Synaptic transmission and the restoration of ion gradients across the cell membrane following depolarisation by action potentials and post-synaptic potentials requires much energy in the form of the molecule ATP (adenosine triphosphate), which is largely generated by oxidative metabolism within the neuron’s mitochondria (Attwell et al. 2010). However, a seminal line of research (Sokoloff et al. 1955; Clarke and Sokoloff 1999) has established that an increase in mental task demand, requiring more neural computations, has no effect on the overall level of cerebral oxygen metabolism across the full brain compared to a rest condition. This can be taken to suggest that the overall level of cerebral metabolism places a hard limit on mental processing, so that any increase in task-related demands on neural computation must be met with a decrease in task-unrelated processing. Taken together with the aforementioned cognitive and neuroimaging findings on the bi-directional limits between attended and unattended processing, specifically in the case of MW and perceptual processing, we propose that the common limiting resource can be directly attributed to the limited amount of neural metabolism that is available to either process in relation to whether it is attended or unattended in the current task.

This account predicts that, as the level of perceptual load in the attended task is increased, an attention-regulated metabolism trade-off mechanism allows for the greater task-induced demand on neural computations to be met with more metabolic energy supply, at the expense of any task-irrelevant processing which suffers a parallel reduction in metabolism levels, therefore resulting in reduced MW. Conversely, given that the brain-wide level of cerebral oxygen metabolism remains the same even when a task involves only a low level of demand on neural computations (e.g. low perceptual load), a “spill over” of available metabolic resources to task-irrelevant processing would be expected (Bruckmaier et al. 2020), and this would allow for increased experiences of MW.

To investigate these predictions, we used broadband near-infrared spectroscopy (BNIRS), specifically developed to measure cerebral oxygen metabolism via the changes in intracellular enzyme marker, the oxidation state of cytochrome c oxidase (oxCCO; Jöbsis 1977; Bale et al. 2016). We placed the BNIRS optodes over the prefrontal cortex ensuring that the central optodes cover medial prefrontal cortex (mPFC) which is considered a main node of the default mode network (DMN). The mPFC node of the DMN was determined to be a suitable region for mind-wandering-related metabolism based on former PET studies (Mazoyer et al. 2001; Raichle et al. 2001) establishing that changes in the level of metabolism and cerebral blood flow in the DMN, including specifically the mPFC, are associated with resting brain states that facilitate mind wandering (vs. brain states that prevent mind wandering, including task states or vegetative states; Laureys et al. 1999; Mazoyer et al. 2001). Other research using fMRI has also specifically associated mPFC activity (deduced from blood flow or BOLD signals) with mind wandering (Mckiernan et al. 2003; McKiernan et al. 2006; Mason et al. 2007; Christoff et al. 2009; Stawarczyk et al. 2011).

Participants performed a visual search task under either low (feature pop-out search) or high (conjunction search) perceptual load conditions. To assess mind wandering participants were prompted at irregular intervals during the task to report whether their thoughts were task unrelated (MW) or task focused (TF). This experience-sampling procedure allowed us to measure the oxCCO levels specifically associated with periods of MW or TF, as well as how the differential oxCCO signal associated with MW versus TF was affected by the level of perceptual load in the task.

In Experiment 1 we tested whether high perceptual load in the task will result in a reduced level of cellular metabolism in mPFC regions associated with MW (vs. TF) during task performance. In Experiment 2 we investigated a further prediction concerning the effects of perceptual load on mind wandering experience, specifically, given that highly-detailed thoughts are expected to place a greater demand on neural metabolism compared to vague- not fully formed- thoughts we tested whether increased perceptual load in a task specifically reduces the experience of detailed rather than vague task-unrelated thoughts.

## Materials and methods

### Experiment 1: Cellular metabolism modulations by MW and perceptual load

#### Participants

We based our sample size calculation on the perceptual load modulation of oxCCO related activity to an unattended visual distractor (Bruckmaier et al. 2020) and estimated that 16 participants would be required to find perceptual load modulations of MW-related activity in the mPFC (assuming 80% power, and a 5% alpha threshold). We recruited some extra participants (total 19) in case any needed to be excluded during data analysis. Indeed, two participants did not report sufficient periods of MW for our within-subject analysis (< 10% of all probes) and had to be excluded from any further analysis. Thus, the final sample size for the rest of the analyses was 17 participants (10 female, age range 18–33), all with normal or corrected to normal vision. This study was approved by the UCL ethics committee, and all participants gave written consent prior to their participation.

#### Stimuli and procedure

**Fig. 1:**
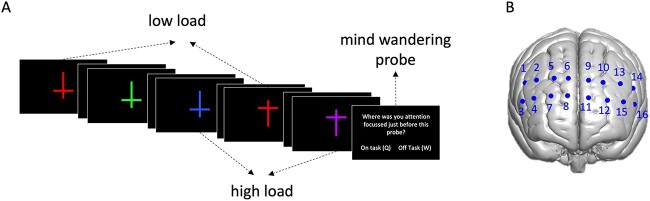
Experiment procedure. A) Schematic (not to scale) depiction of the task procedure. Perceptual load varied through instruction (low load: feature search—detect any red cross, high load: conjunction search—detect upright purple or inverted blue cross. The stream of crosses was interrupted at irregular intervals with MW probes. B) Average position of the 16 optode channels.

Participants performed a rapid serial visual presentation (RSVP) task with a feature search (low load) or conjunction search (high load) manipulation (Schwartz et al. 2005; Carmel et al. 2011; Ohta et al. 2012; Bruckmaier et al. 2020): Each RSVP stream started with the presentation of a white fixation cross on a black background for 1,000 ms which was followed by the presentation of a stream of crosses in the centre of the screen (each 0.08° × 0.06° of visual angle; see Fig. 1A). Each cross was presented for 250 ms and followed by 500 ms blank ISI. The crosses were either oriented upright or inverted and were colored either blue (0 115 255), green (0 255 0), yellow (255 255 0), purple (160 32 240), red (255 0 0), or brown (156 102 31). The order of crosses was pseudo-randomized with the constraints that no two targets could be presented straight after each other and that the first item of each stream was never a target.

The participants were required to press a button in response to target crosses, which were defined before each RSVP stream. For the high load condition, targets were defined based on color and orientation (i.e. an upright purple or an inverted blue cross), while in the low load condition targets were defined based on color alone, irrespective of orientation (i.e. any red cross). Non-targets could be any color/orientation combination apart from the target color (low load) or target color/orientation combination (high load). This was determined randomly with the only constraint that the opposite target/orientation combination to the high load targets (i.e. upright blue or inverted purple crosses) was twice as likely as any other color/orientation combination in both the high and low load streams (in order to keep the streams visually equivalent). Perceptual load was thus varied only through the task instructions. Each RSVP stream had 320 cross stimuli (12.5% of which were targets). Participants completed 12 RSVP streams (approximately 4 min per stream) 6 of high (conjunction search, 6 streams) and 6 of low (feature search) perceptual load arranged in an ABBABAAB pattern, each followed by a 30 s break during which participants received automated feedback on their performance. The first 3 participants completed six 8-min streams instead (3 in each of the perceptual load conditions, arranged in an ABBABA pattern). Five seconds before each stream instructions indicating the next targets that appeared on the screen.

In addition to the visual search task, participants had to respond to occasional thought probes throughout the task. The thought probes consisted of a written question on screen, prompting “Where was your attention focused just before this probe?” with two possible answers “on task” and “off task”. Responses were given by button press without time limit (buttons “Q” for “on task” and “W” for “off task”). After the participant’s response, the RSVP stream continued. Five thought probes (or 10 for the first 3 subjects^1^) were presented per each 4-min stream at pseudo-random intervals ranging from 20 s to 75 s (average across all trials 48 s), resulting in a total of 60 thought probes, 30 per each of the load conditions. The only other constraint regarding the placement of the thought probes was that they could never appear directly before or after an RSVP target. The randomized patterns of time intervals between probes were kept consistent between every two successive streams of different load levels.

Before the start of this experiment, participants were instructed to answer honestly and informed that “off-task” responses would not be penalized in any way. Moreover, they were given examples of “on-task” and “off-task” thoughts (e.g. “What will I have for dinner?” as off-task thought, “My target is any red cross” as on-task thought). They then completed two short practice streams (one of each load condition, 30 s per stream) to familiarize themselves with the task and were given the opportunity to ask questions.

#### Instrumentation

We used a BNIRS system specifically developed to measure the oxidation state of the enzyme cytochrome c oxidase (oxCCO), which is the terminal acceptor in the mitochondrial electron transfer chain. Since the overall concentration of CCO does not change within the timeframe of a typical experiment (i.e. hours), the ratio of oxidized and reduced CCO can be used as an intracellular marker of mitochondrial oxygen metabolism. By using a broadband light spectrum from 780–900 nm, BNIRS has been shown to be able to successfully isolate the oxCCO signal (which has a broad absorption peak at 830 nm) from the signal elicited by oxygenated (HbO_2_) as well as deoxygenated hemoglobin (HHb) (Jöbsis 1977; Bale et al. 2016). This measure has been validated using a range of alternative, invasive methods for measuring metabolism levels in humans and animals (Cooper and Springett 1997; Peeters-Scholte et al. 2004; Bainbridge et al. 2014; Kaynezhad et al. 2019; Mitra et al. 2019).

The multi-channel BNIRS system used in this study had 4 source fibers and 10 detector fibers which were arranged in two rows of five detectors with the 4 source fibers in between, and a source detector separation of 30 mm. Overall, this resulted in 16 measurement channels (see Fig. 1B). The optodes were fixed in a custom array, which was placed over frontal cortex approximately 6 cm above the nasion, and optode positions were digitized using a Patriot Digitiser (Polhemus), with the inion, nasion, left and right peri-auricular points, O1, O2, and vertex serving as reference points (based on 10/20 electrode system). The digitized positions were converted to MNI coordinates using NIRS SPM (Ye et al. 2009; see Supplementary Table 1).

#### Pre-processing

We transformed the attenuation data into chromophore concentration changes (oxCCO, HbO_2_, and HHb) by applying the UCLn-algorithm using the modified Beer-Lambert law, assuming a differential pathlength factor of 6.26 and its wavelength dependence (Phan et al. 2016). Since the streams in this experiment lasted for 4–8 min, low-frequency fluctuations in the data were likely to contain task-related information. Therefore, a lowpass filter was applied with a cut-off frequency of 0.08 Hz, preserving low-frequency components of the signal. To remove low-frequency changes that are not induced by the periodicity of the long task blocks and reflect noise of various physiological or technological origin, the data was de-trended by subtracting a linear fit between the mean of the 10 s preceding and following each stream. Motion artifacts were corrected using a wavelet-based algorithm with an IQR parameter of 1.5 (Molavi and Dumont 2012). Since this algorithm could not correct for step-like motion artifacts (where the signal never returns to the same level as before the artifact vs. a spike-like motion artifact where the signal returns to the same level as pre-artifact), we inspected the data manually and excluded the full stream if it contained step-like motion artifacts, since this would affect the detrending procedure for the entire stream. This was done on a channel-by-channel basis (given that each optode could undergo some movement independently of another). Following this removal, one participant (for which set up issues were recorded) had no streams remaining in one of the four conditions in channels 1–4; 9–11 and 13 and so their data was removed from those channels; on this basis, data from a further participant had to be excluded from channels 2, 4, 5, and another from channels 1 and 14. In addition, any stream in which participants correctly responded to fewer than 50% of targets were also removed (a total of 3 streams across all participants, i.e. 0.6% of all data analyzed).

Channels were assigned to Brodmann areas for each subject based on their MNI coordinates in order to minimize the number of statistical comparisons and thus the likelihood of false positives. Group-means of each channel’s MNI coordinates and Brodmann area allocation can be seen in Supplementary Table 1. Regions of interest (ROI) were based on previous PET studies (Laureys et al. 1999; Mazoyer et al. 2001; Raichle et al. 2001) that established that changes in the level of metabolism and cerebral blood flow in the DMN (including specifically the mPFC, covering medial parts of BA 9 and BA 10) are associated with “resting” brain states (compared to task states or vegetative states) during which more MW is likely to occur (Mason et al. 2007).

#### Statistical analyses

For each participant, the mean oxCCO signal across the 15 s interval preceding each thought probe was calculated for each of the four experiment conditions of perceptual load (low vs. high) and MW probe report (MW vs. TF), in each of the ROIs. 2×2 repeated measures ANOVAs with the factors of perceptual load (low/high) and MW report (MW/TF) were run on the oxCCO signal in the 4 ROIs: left and right BA9, and left and right BA10. To narrow down the spatial region of the effect, significant effects were followed with the same 2×2 repeated measures ANOVAs run on the channels within these BA areas. To ensure that any effects found were not driven by a few individuals disproportionally affecting the mean signals, and to identify the specific time periods of the effects, all significant effects were followed with a bootstrap resampling analysis (1,000 iterations, balanced; Efron and Tibshirani 1993). Effects were considered significant if 95% or more of the bootstrap iterations fell above/below zero (i.e. *P* < 0.05) for 10 or more consecutive 1 s samples. For the main effects, or simple main effects (in the case of significant interactions in the analyses of the means) the bootstrap resampling was run on the difference between the two levels of the variable (perceptual load or MW report) for each second preceding the probe during the 15 s period. For interactions, bootstrap resampling was run to compare the difference between the simple main effects of MW report (i.e. the differential oxCCO signal increase during MW versus TF) in the low load and the high load conditions for the second-by-second periods specified above.

### Experiment 2: Perceptual load modulations of MW levels of detail

#### Participants

Experiment 2 was programmed and hosted on the online platform Gorilla (https://gorilla.sc), and participants were recruited using Prolific (https://www.prolific.co). To ensure our study had sufficient power to detect effects on the level of MW detail we based our sample size calculation on the effect on MW “vagueness” (which we considered most similar to our measure of the level of detail) found in Seli et al. (2017), for another within-subject factor (of intention), since perceptual load effects on MW detail have not as yet been studied). Based on the effect size of d = 0.47 (corresponding to Cohen’s F = 0.25) and a power of 80%, a sample of 38 participants is sufficient to find a significant effect at an alpha level of 0.05. Since our study was conducted online and may thus be noisier than a study conducted in a lab environment, we decided on an increased sample size of 44 participants. Participants who did not report any MW for at least one of the load conditions could not be included and were replaced until the full sample size was reached. This led to a total of 19 participants that had to be excluded and replaced with new participants. In addition, we excluded and replaced any participant for whom the number of FAs was equal to or greater than 90% of the number of correct hits in the high load condition, which indicates that they may have based their responses on color alone (rather than conjunction of color and orientation). This resulted in the exclusion and replacement of 4 participants. The final sample consisted of 44 participants (27 female, age range 18–34) with normal or corrected to normal vision. This study was approved by the UCL ethics committee, and all participants gave written consent prior to their participation.

#### Stimuli and procedure

The same RSVP cross task as in Experiment 1 was used with the following modifications: The task was presented in a browser window. The colored crosses were presented in a large, black square (400 × 400 pixels, approximately 4.8 × 4.8° visual angle for participants who are set at 60 cm from the screen on a light gray background. Participants completed a total of 5 streams of 320 stimuli each, with 5 thought probes per stream (as per Experiment 1) in each of the load conditions, resulting in a total of 25 thought probe responses per load condition. Thought probes were followed by another screen with a follow-up question asking participants to rate the level of detail of their thoughts on a scale from 0 to 100. This follow-up screen appeared regardless of the participants’ response to the MW question (in order to avoid a strategy of always reporting task focus in order to avoid the extra question, and thus speed up the experiment).

#### Statistical analyses

Task RT, misses, and false alarms were calculated as a function of perceptual load and MW report and were examined by 2×2 repeated measures ANOVAs. To analyze the effect of perceptual load on detail, detail ratings following a MW report were classified into “high-detail” or “low-detail” according to whether ratings were greater or smaller than 50, respectively. The corresponding rates for detailed vs. non-detailed MW were then entered into a 2×2 repeated measures ANOVA with the additional factor of load to examine whether MW detail varied as a function of perceptual load.

## Results

### Experiment 1: Cellular metabolism modulations by MW and perceptual load

#### Behavioral

**Fig. 2:**
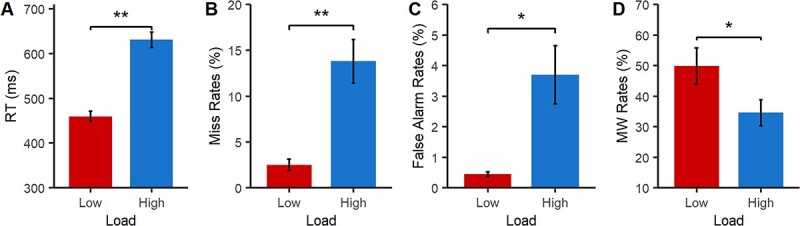
Task performance and MW rates as a function of perceptual load. Means (±SEM) are shown for A) RT. B) Miss rates. C) False alarm rates. D) MW rates. ^*^P < 0.05, ^*^^*^P < 0.001.

Figure 2 shows the behavioral results. As expected, high perceptual load resulted in increased RTs (*t*(16) = 17.41, *P* < 0.001, *d* = 2.88), miss rates (*t*(16) = 5.50, *P* < 0.001, *d* = −1.58), and false alarm rates (*t*(16) = 3.49, *P* = 0.003, *d* = 1.14) compared to the low load conditions (Fig. 2A–C), thus confirming the efficacy of the perceptual load manipulation. Moreover, high perceptual load significantly reduced MW rates compared to low perceptual load, as predicted (*t*(16) = −2.47, *P* = 0.025, *d* = −0.71; see Fig. 2D).

#### oxCCO analysis

**Fig. 3:**
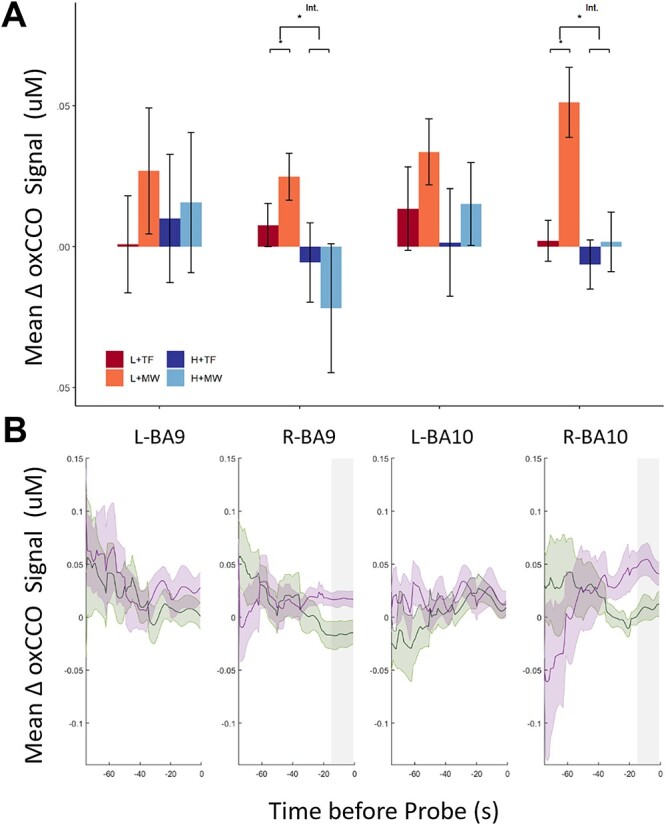
Mean and time series of oxCCO signal change prior to MW report probes: ROI results. A) Mean (±SEM) oxCCO signal change compared to baseline per condition (high/low load × MW/TF) in BA9 and BA10 (left/right), during the 15 s preceding each thought probe. B) Time series for the mean (±SEM) oxCCO signal for MW-TF in low and high load for the full period preceding a probe. Gray bars indicate the significant time period of the interaction identified by the bootstrapping analysis. ^*^P < 0.05, ^*^^*^P < 0.001.

The oxCCO results for the ROI analyses can be seen in Fig. 3. The 2×2 ANOVAs revealed a significant main effect of MW report, reflecting increased oxCCO levels during time periods preceding MW reports versus TF reports, in right BA10 (*F*(1,11) = 7.77, *P* = 0.018, *η_p_^2^* = 0.41), and a similar trend of marginal significance in left BA10 (*F*(1,12) = 4.56, *P* = 0.054, *η_p_^2^* = 0.28). There were no main effects of MW report in the other BAs (*F*(1,15) = 1.24, *P* = 0.284, for left BA9 and *F*(1,16) = 0.004, *P* = 0.949, for the right BA9). A main effect of perceptual load was found in right BA10, reflecting that the oxCCO signal was significantly increased during low (vs. high) load conditions (*F*(1,11) = 9.95, *P* = 0.009, *η_p_^2^* = 0.47). No significant main effects of perceptual load were found in the other areas (all *F* < 3.46, *P* > 0.081). Importantly, there was a significant interaction between perceptual load and MW report in right BA10 (*F*(1,11) = 15.22, *P* = 0.002, *η_p_^2^* = 0.58), and in right BA 9 (*F*(1,16) = 4.89, *P* = 0.042, *η_p_^2^* = 0.23), (*F* < 1.30; *P* > 0.272 for the two left regions). This terminative interaction reflected that MW (vs. TF) was associated with increased oxCCO signal when perceptual load was low (right BA10: *t*(11) = 3.81, *P* = 0.003, *d* = 1.10; right BA9: *t*(16) = 2.73, *P* = 0.014, *d* = 0.66), but not during high perceptual load (right BA10: *t*(11) = 0.80, *P* = 0.440; right BA9: *t*(16) = −1.29, *P* = 0.214), see Fig. 3. The terminative interaction also clarified that the increase in oxCCO in the low (vs. high) load conditions was just attributed to simple main effect of increased oxCCO in MW periods (right BA10: *t*(11) = 4.00, *P* = 0.002, *d* = 1.15; right BA9: *t*(16) = 2.16, *P* = 0.046, *d* = 0.53), as no simple main effect of load was found in TF periods (right BA10: *t*(11) = 0.99, *p* 0.343; right BA9: *t*(16) = 1.02, *P* = 0.322). Bootstrap analyses confirmed that all the significant main effects and interactions were significant throughout the whole 15 s preceding a probe.

**Fig. 4:**
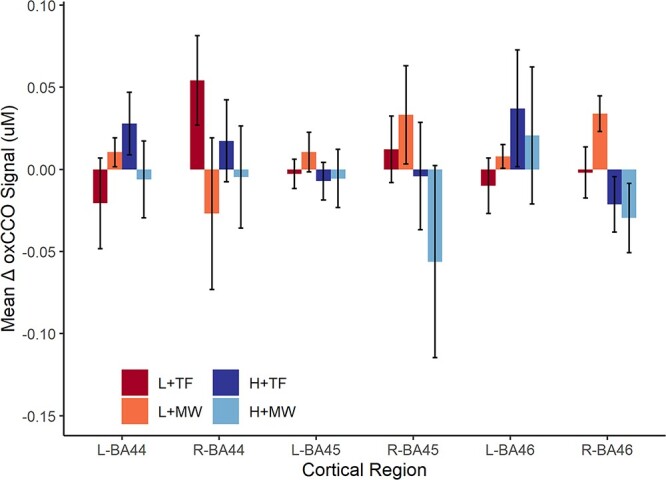
Mean oxCCO signal change prior to MW report probes: BAs outside the ROIs. Mean (±SEM) oxCCO signal change compared to baseline per condition (high/low load x MW/TF) in BA 44, BA45 and BA46 (left/right) during the 15 s preceding each thought probe.

Figure 4 shows the data for the other regions measured, that were clearly outside mPFC. None of the trends shown reached statistical significance (all *F’s* < 4.56, *p_FDR_* > 0.270, and all F’s < 7.20, *p_FDR_* > 0.094 for the main effects of MW, and load respectively, all *F’s* < 5.99, *p_FDR_* > 0.136 for the interaction between load and MW).

#### oxCCO—Channel analysis

Since BA9 and BA10 are large and extend laterally beyond the mPFC, the analysis was repeated for the individual channels with mean coordinates that fall within the significant ROIs (channels 2, 5, 6, and 8, see coordinates in Supplementary Table 1 and diagram in Fig. 1B), in order to localize the effect on a more fine-grained spatial scale. While channels 6 and 8 were close to the midline, channels 2 and 5 were positioned more laterally.

**Fig. 5:**
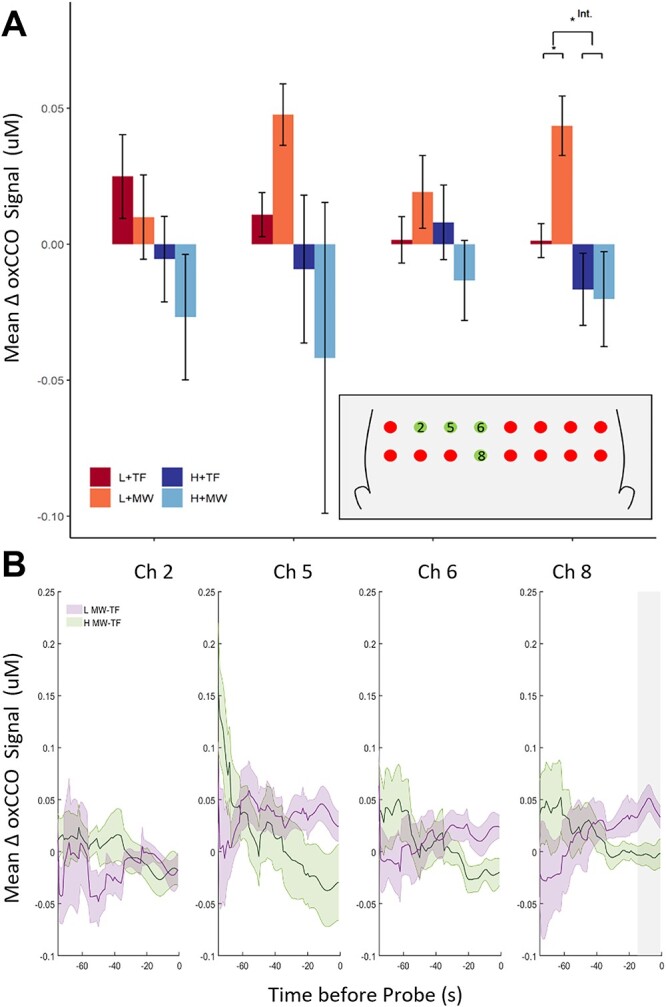
Mean and time series of oxCCO signal change prior to MW report probes: ROI channel results. A) Mean (±SEM) oxCCO signal change compared to baseline per condition (high/low load x MW/TF) in channels 2, 5, 6 and 8, during the 15 s preceding each thought probe. B) Time series for the mean (±SEM) oxCCO signal for MW-TF in low and high load for the full period preceding a probe. The gray bar indicates the significant time period of 15 sec identified by the bootstrapping analysis for the interaction. ^*^P < 0.05, ^*^^*^P < 0.001.

There was no main effect of MW report in any channel (all *F* < 3.83, *P* > 0.067), but there was a significant main effect of load in channel 8 (*F*(1,16) = 10.14, *P* = 0.006, *η_p_^2^* = 0.39), reflecting higher activity in low than high perceptual load (see Fig. 3). The main effect of load failed to reach significance in the other channels (all *F* < 3.53, *P* > 0.081). The main effect of load was qualified by an interaction between MW report and perceptual load in Channel 8 (*F*(1,16) = 20.79, *P* < 0.001, *η_p_^2^* = 0.57). As can be seen in Fig. 5 this interaction mirrored the directions observed in the BA analysis indicating a significant increase in the oxCCO signal during MW compared to TF periods in low perceptual load, (*t*(16) = 3.56, *P* = 0.003, *d* = 1.12), but not high perceptual load (*t*(16) = −0.34, *P* = 0.735). The interaction was not significant in the other channels of interest, however, there was a marginally significant trend in channel 6, which is the other midline channel (channel 6: *F*(1,16) = 4.08, *P* = 0.060; cf. channel 5: *F*(1,16) = 2.38, *P* = 0.144; channel 2: *F*(1,16) = 0.03, *P* = 0.863). Bootstrap analyses confirmed that all the significant main effects and interactions were significant throughout the whole 15 s preceding a probe.

### Experiment 2: Perceptual load modulations of MW levels of detail

Experiment 1 showed that a manipulation of high perceptual load in the task results in reduced oxygen metabolism in mPFC regions associated with MW reports. This pattern is in support of our account of shared metabolism resources between perception and MW. In Experiment 2, we further investigated the nature of the MW reports, specifically their level of detail, since we found reduced metabolism levels in the region of the mPFC, which previous research has associated with the level of thought detail (Sormaz et al. 2018). Since highly-detailed thoughts are expected to involve a greater demand on neural metabolism than vague thoughts we hypothesized that highly detailed MW would be more affected by perceptual load than MW involving only vague thoughts.

#### Behavioral

**Fig. 6:**
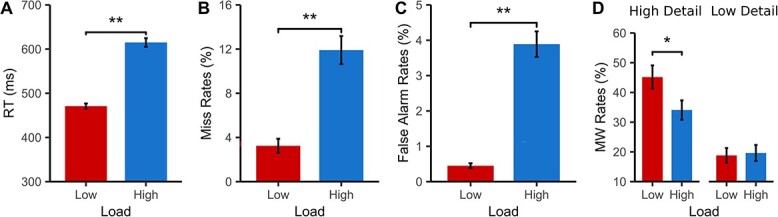
Task performance and MW rates of high or load detail as a function of perceptual load. Means (±SEM) are shown for: A) RT. B) Miss rates. C) False alarm rates. D) MW rates of high or low detail. ^*^P < 0.05, ^*^^*^P < 0.001.

Figure 6 shows the results of Experiment 2. As in Experiment 1, high perceptual load increased the RT (*t*(43) = 20.57, *P* < 0.001, *d* = 3.10), increased miss rates (*t*(43) = 8.53, *P* < 0.001, *d* = 1.29) and increased false alarm rates (*t*(43) = 10.55, *P* < 0.001, *d* = 1.59), see Fig. 6A–C). Importantly, as before, the rate of MW reports was significantly reduced under high (vs. low) perceptual load (*t*(43) = 2.85, *P* = 0.007, *d* = 0.46).

A 2x2 repeated measures ANOVA with the factors of load and detail was run (see Fig. 6D). As in Experiment 1, a main effect of load was observed (*F*(1,43) = 8.04, *P* = 0.007, *η_p_^2^* = 0.157), which reflected significantly reduced levels of MW (across their level of detail) in high compared to low load. There was no main effect of detail (*F*(1,43) = 0.08, *P* = 0.784, *η_p_^2^* = 0.002), but there was a significant interaction between detail and load (*F*(1,43) = 11.89, *P* = 0.001, *η_p_^2^* = 0.217). As can be seen in Fig. 6D, this interaction reflected that, highly detailed MW was significantly reduced in the high load compared to the low load conditions (*t*(43) = 4.09, *P* < 0.001, *d* = −0.616), while MW of low detail was unaffected by load (*t*(43) = 0.33, *P* = 0.740), in line with our prediction.

## Discussion

The present study used BNIRS to directly measure an intra-cellular, mitochondrial marker of cellular metabolism (Jöbsis 1977; Bale et al. 2016) related to MW reports sampled throughout a visual attention task with varying levels of perceptual load. The results allowed us to directly establish a cerebral metabolism account of the relationship between MW and perceptual task demands. The level of cellular oxygen metabolism related to MW (as opposed to TF reports) in mPFC (specifically within the medial regions of right BA9 and right BA10) was found to be significantly modulated by the level of perceptual load in an attended visual perception task. In task conditions of low perceptual loadthere was a significant increase in right mPFC metabolism levels during periods of MW compared to periods of TF. Conversely, higher perceptual load in the task resulted in reduced metabolism levels related to MW (vs. TF) in the right mPFC. Importantly, these modulations were found in an orthogonal perceptual load (low vs. high) and MW report (MW vs. TF) design where MW (vs. TF) related metabolism levels could be attributed to the increased metabolism signal specifically due to MW as compared to TF in either of the perceptual load levels, and perceptual load did not involve any change to the external stimuli- just to their perceptual processing demands. Thus, the modulation in MW-related metabolism by perceptual load could not be attributed to any change in the external stimulation. In addition, participants’ rate of MW reports were reduced during high load conditions, consistent with previous behavioral reports (Forster and Lavie 2009; Morris et al. 2020). A follow-up experiment clarified that it was detailed MW that was reduced with high perceptual load, while MW of low detail remained unaffected.

Overall, these findings fit with our common resource sharing account that proposes oxygen metabolic energy as a capacity-limiting resource for ongoing parallel processing of perceptual information and internal thought. Since the amount of cerebral oxygen metabolism at any given time is limited (Sokoloff et al. 1955; Clarke and Sokoloff 1999), an attentional balancing mechanism regulates oxygen metabolism resources across the brain to accommodate perceptual task processing demands and DMN activity supporting mind-wandering. Crucially, this balance depends on the neural processing demands in the attended task which varies with the levels of its perceptual load (i.e. the demands placed on perceptual processing). Load theory of attention states that higher perceptual load in an attended task reduces resources for any task-irrelevant processing due to increased resource consumption associated with attended, task-related processing. Conversely, attended tasks of low perceptual load leave spare capacity available, which is predicted to spill over to task-irrelevant processes (Lavie 1995; Lavie 2005). Our previous research, using the same perceptual load manipulation as used here, demonstrated such a trade-off of cerebral metabolism levels between attended and unattended visual stimuli within visual cortex. This finding supported the account that limited cerebral metabolism levels are the underlying cause of the limited capacity effects seen in perceptual processing (Bruckmaier et al. 2020). Here we extend the evidence for this claim to the metabolism increases in the frontal cortex that are related to task-unrelated thoughts (i.e. MW), and demonstrate that perceptual load modulates them during task performance.

Our results also provide a new line of evidence for previous claims concerning the functional role of DMN metabolism levels (especially in the mPFC) in relation to the processing demands imposed by the attended task. In their seminal study, which established the DMN based on its metabolic properties at rest, Raichle and colleagues (Raichle et al. 2001) have previously speculated that DMN activity at rest may serve a range of functions associated with “non-urgent” evaluations of the external as well as internal environments. They further propose that these evaluations (and associated activity in the DMN) may be suspended during task states when additional neural computations elsewhere place increased demand on our metabolic capacity. Our results serve as a direct demonstration that external task demands indeed modulate the metabolism levels of the mPFC node of the DMN. Moreover, we have directly related levels of metabolic signal to people’s reported experience of MW (as opposed to TF) in further support of Raichle’s work based on metabolism levels in resting state periods. The latter finding is consistent with findings based on the BOLD response that have also linked increased DMN activity to MW reports during the task period (Christoff et al. 2009; Stawarczyk et al. 2011; Konu et al. 2020) and suggest these are driven by levels of underlying metabolism.

Since Raichle’s initial suggestion of the DMN, much work has expanded on the role of the DMN in cognition. A range of functions have been suggested, all of which can be broadly unified as forms of cognition that are unrelated or only loosely related to external events at the time of MW (see Smallwood et al. 2021a). These include, for example, thoughts involving autobiographical memory and “mental time travel” (e.g. Svoboda et al. 2006; Addis et al. 2007), or reflections about the self (Kelley et al. 2002) and others (e.g. theory of mind, or more generally higher order social cognition, see Mars et al. 2012). Experience sampling studies have shown that MW often involves these type of thoughts (Ho et al. 2020; Smallwood et al. 2021b). Importantly, research has shown functional specialization of regions and sub-networks within the DMN (Andrews-Hanna et al. 2010; DiNicola et al. 2020). The dorsal mPFC region associated with increased metabolic signal during MW that we report was previously associated with self-referential thoughts about one’s traits, abilities, and attitudes, or one’s current states, e.g. emotional feelings (Gusnard et al. 2001; Johnson et al. 2002; Ochsner et al. 2004; Andrews-Hanna et al. 2010), as well as judgments about others (Gallagher et al. 2000; D'Argembeau et al. 2007). It is plausible therefore that the MW reports in our study involved similar functions. However, since we have merely classified the reports into MW or TF, and only examined their level of detail, future research is needed to clarify any functional role of the increased metabolism levels in relation to the MW reported in our study. Given also that BNIRS cannot detect metabolism levels in deeper cortical layers (beyond ~2 cm from the scalp; e.g. Patil et al. 2011), it is plausible that our findings reflect the tip of the iceberg of a more extensive modulation, including for example also more ventral mPFC regions.

Importantly, our metabolism trade-off account can explain the widely-observed phenomenon of “perceptual decoupling” (Schooler et al. 2011; Smallwood and Schooler 2015). Perceptual decoupling has been demonstrated with reports of reduced ERPs and BOLD responses related to perception during MW (Braboszcz and Delorme 2011; Kam et al. 2011; Smallwood et al. 2012; Baird et al. 2014; Denkova et al. 2018; Cohen et al. 2022; Zhang et al. 2022). Many of these have included EEG components associated with early sensory processes (e.g. N1, P1) thus extending earlier suggestions (Smallwood 2010) that MW leads to resource competition for access to the frontoparietal “global workspace” thought to mediate perceptual awareness (e.g. Dehaene and Changeux 2005) to show wide-spreading modulations involving reduced sensory (potentially unconscious) processing of external (visual and auditory) input. Our research suggests that these interactions between MW and perceptual processing are in fact bi-directional, with reduced MW-related metabolism levels observed in the mPFC when perceptual demand in a visual attention task is increased. Taken together, these modulation patterns suggest that perceptual decoupling may occur as a consequence of reduced metabolism availability for perception. Conversely, when the primary task is demanding on perception (high perceptual load), the frequency of MW and the levels of the underlying metabolic activity are reduced because of the increased allocation of metabolic energy to cortical regions sub-serving perceptual processing. This interpretation is particularly supportive of the view of perceptual decoupling as a consequence of competition for limited resources due to the mentally demanding nature of MW (Franklin et al. 2013; Smallwood 2013) and points to the limit in cerebral metabolism supply as the underlying limited resource. Although our interpretation provides a unified account for a variety of previous findings (Braboszcz and Delorme 2011; Kam et al. 2011; Smallwood et al. 2012; Baird et al. 2014; Denkova et al. 2018; Cohen et al. 2022; Zhang et al. 2022), including both EEG and BOLD modulations, we note that since the BOLD signal is affected not only by neural metabolism but also by the rate of cerebral blood flow which is also coupled with both local (glutamate) and global neurotransmitter signaling processes (Attwell and Iadecola 2002), it is only an indirect marker of cerebral metabolism levels, and its interpretation as such needs to be cautioned (Logothetis 2008). Therefore, a demonstration of whether perceptual decoupling during MW results in reduced metabolism levels related to perception in sensory cortices should be an important direction for future research.

Our findings show that high perceptual load is not only associated with reduced metabolism in the mPFC node of the DMN, but also with a specific reduction in the rate of highly detailed MW, whereas MW with low detail levels appears to proceed unaffected by perceptual load. This is consistent with previous findings that mPFC activity is particularly sensitive to the level of detail in spontaneous thoughts (Sormaz et al. 2018). It is also consistent with our and others’ (Franklin et al. 2013) accounts of resource sharing between MW and perception. Detailed MW is expected to require a higher level of metabolism, and therefore is more likely to be reduced when the perceptual demands of the task are increased, and metabolism levels in the mPFC are low.

Finally, while here we have demonstrated the implications of increased perceptual task demands for mPFC levels of metabolism in relation to MW, our account of the mutually limiting relationship of perception and MW can be generalized to accommodate a wider range of tasks and mental activities that compete for limited cerebral metabolism. For instance, DMN activity has been shown to be reduced with increased processing demands in various tasks including, in addition to perceptual load (Ohta et al. 2012), also visual working memory, executive function, visual motion discrimination, and auditory discrimination (McKiernan et al. 2003; McKiernan et al. 2006; Mason et al. 2007; Singh and Fawcett 2008; Sormaz et al. 2018). These tasks have also been shown to reduce the frequency of MW. However, these previous demonstrations were all somewhat indirect, since none of these studies used an experience sampling approach while recording brain activity, thus not allowing direct associations between MW episodes and DMN levels of activity. A direct demonstration that tied perceptual decoupling in a visual working memory task to brain activity related to MW experience, as well as its level of detail, has recently been reported (Turnbull et al. 2019). The present study further adds a direct link between DMN metabolism levels related to MW reported during task performance, and the critical role of task processing demands in determining both. Our shared-metabolism account leads to the prediction that increased mental processing demands in a variety of tasks would result in similar reductions in DMN metabolism levels as those shown here. Similarly, brain activity related to additional mental processes, both internal (e.g. to include task-related thoughts) and external (e.g. to include multisensory processing), is also expected to adhere to similar capacity limits resulting in metabolism trade-off between attended and unattended processing in the related brain regions as task demands are increased. Future research testing these predictions should prove important for a comprehensive neurobiological account of attention and capacity limits in cognition.

## Conclusion

By using BNIRS to directly measure an intra-cellular marker of cerebral metabolism in the brain during task performance, we established that the magnitude of the metabolic response associated with MW critically depends on the level of perceptual load in the currently attended task. Increased perceptual load in the task resulted in reduced mPFC metabolism levels during periods in which participants reported MW, as well as reduced frequency of MW overall, and reduced levels of detail for any remaining experiences of MW. On the other hand, low perceptual load resulted in higher mPFC metabolism levels associated with MW, and the reported MW experiences also involved highly detailed thoughts. These results support a neurobiological account attributing the mutually limiting relationship of MW and perceptual processing to general limits on brain-wide cerebral metabolism. They also demonstrate that when spare metabolic resources are available under conditions of low perceptual load, these can spill over not only to the processing of external stimuli outside the focus of attention, as previously shown (Bruckmaier et al. 2020), but also to internally focussed processes such as MW. Overall, our approach demonstrates an important bridge between the concept of capacity-limited cognitive processing and the corresponding limits on metabolic energy supply for neural computation. As such, it allows for a better understanding of the neurobiological mechanisms underlying attention and capacity limits in cognition.

## Supplementary Material

SupplementaryMaterials_tgad021

## Footnotes

1 The mean MW rates for the first three subjects were 46% and 39% in low and high load, respectively, and these rates were within 1 standard deviation of the mean rates of the last 14 subjects (50% (SD = 0.25) and 34% (SD = 0.18) for low and high load, respectively). Thus, this minor methodological difference did not seem to affect the results.

## References

[ref1] Addis DR, Wong AT, Schacter DL. Remembering the past and imagining the future: common and distinct neural substrates during event construction and elaboration. Neuropsychologia. 2007:45(7):1363–1377. 17126370 10.1016/j.neuropsychologia.2006.10.016 PMC1894691

[ref2] Andrews-Hanna JR, Reidler JS, Sepulcre J, Poulin R, Buckner RL. Functional-anatomic fractionation of the brain’s default network. Neuron. 2010:65(4):550–562. 20188659 10.1016/j.neuron.2010.02.005 PMC2848443

[ref4] Attwell D, Iadecola C. The neural basis of functional brain imaging signals. Trends Neurosci. 2002:25(12):621–625. 12446129 10.1016/s0166-2236(02)02264-6

[ref5] Attwell D, Laughlin SB. An energy budget for signaling in the grey matter of the brain. J Cereb Blood Flow Metab. 2001:21(10):1133–1145. 11598490 10.1097/00004647-200110000-00001

[ref6] Attwell D, Buchan AM, Charpak S, Lauritzen M, Macvicar BA, Newman EA. Glial and neuronal control of brain blood flow. Nature. 2010:468(7321):232–243. 21068832 10.1038/nature09613 PMC3206737

[ref7] Bainbridge A, Tachtsidis I, Faulkner SD, Price D, Zhu T, Baer E, Broad KD, Thomas DL, Cady EB, Robertson NJ, et al. Brain mitochondrial oxidative metabolism during and after cerebral hypoxia-ischemia studied by simultaneous phosphorus magnetic-resonance and broadband near-infrared spectroscopy. NeuroImage. 2014:102:173–183. 23959202 10.1016/j.neuroimage.2013.08.016 PMC4229502

[ref8] Baird B, Smallwood J, Lutz A, Schooler JW. The decoupled mind: mind-wandering disrupts cortical phase-locking to perceptual events. J Cogn Neurosci. 2014:26(11):2596–2607. 24742189 10.1162/jocn_a_00656

[ref9] Bale G, Elwell CE, Tachtsidis I. From Jöbsis to the present day: a review of clinical near-infrared spectroscopy measurements of cerebral cytochrome-c-oxidase. J Biomed Opt. 2016:21(9):091307. 27170072 10.1117/1.JBO.21.9.091307

[ref11] Braboszcz C, Delorme A. Lost in thoughts: neural markers of low alertness during mind wandering. NeuroImage. 2011:54(4):3040–3047. 20946963 10.1016/j.neuroimage.2010.10.008

[ref12] Bruckmaier M, Tachtsidis I, Phan P, Lavie N. Attention and capacity limits in perception: a cellular metabolism account. J Neurosci. 2020:40(35):6801–6811. 32747442 10.1523/JNEUROSCI.2368-19.2020 PMC7455219

[ref13] Carmel D, Thorne JD, Rees G, Lavie N. Perceptual load alters visual excitability. J Exp Psychol Hum Percept Perform. 2011:37(5):1350–1360. 21728464 10.1037/a0024320

[ref14] Christoff K, Gordon AM, Smallwood J, Smith R, Schooler JW. Experience sampling during fMRI reveals default network and executive system contributions to mind wandering. Proc Natl Acad Sci U S A. 2009:106(21):8719–8724. 19433790 10.1073/pnas.0900234106 PMC2689035

[ref15] Clarke D, Sokoloff L. Circulation and energy metabolism of the brain. In: Siegel G, Agranoff B, Albers R, Fisher S, Uhler M, editors. Basic neurochemistry: molecular, cellular and medical aspects. 6th ed. Philadelphia: Lippincott-Raven; 1999. pp. 637–669

[ref16] Cohen D, Nakai T, Nishimoto S. Brain networks are decoupled from external stimuli during internal cognition. NeuroImage. 2022:256:119230. 35460919 10.1016/j.neuroimage.2022.119230

[ref17] Cooper CE, Springett R. Measurement of cytochrome oxidase and mitochondrial energetics by near-infrared spectroscopy. Philos Trans R Soc Lond B Biol Sci. 1997:352(1354):669–676. 9232854 10.1098/rstb.1997.0048 PMC1691958

[ref18] D'Argembeau A, Ruby P, Collette F, Degueldre C, Balteau E, Luxen A, Maquet P, Salmon E. Distinct regions of the medial prefrontal cortex are associated with self-referential processing and perspective taking. J Cogn Neurosci. 2007:19(6):935–944. 17536964 10.1162/jocn.2007.19.6.935

[ref19] Dehaene S, Changeux JP. Ongoing spontaneous activity controls access to consciousness: a neuronal model for inattentional blindness. PLoS Biol. 2005:3(5):0910–0927. 10.1371/journal.pbio.0030141 PMC1074751 15819609

[ref20] Denkova E, Brudner EG, Zayan K, Dunn J, Jha AP. Attenuated face processing during mind wandering. J Cogn Neurosci. 2018:30(11):1691–1703. 30024329 10.1162/jocn_a_01312

[ref21] DiNicola LM, Braga RM, Buckner RL. Parallel distributed networks dissociate episodic and social functions within the individual. J Neurophysiol. 2020:123(3):1144–1179. 32049593 10.1152/jn.00529.2019 PMC7099479

[ref22] Efron B, Tibshirani RJ. An introduction to the bootstrap. An Introd to BootstrapMonographs Stat Appl Probab. 1993:57(1):436.

[ref23] Forster S, Lavie N. Harnessing the wandering mind: the role of perceptual load. Cognition. 2009:111(3):345–355. 19327760 10.1016/j.cognition.2009.02.006 PMC2706319

[ref24] Franklin MS, Mrazek MD, Broadway JM, Schooler JW. Disentangling decoupling: comment on Smallwood (2013). Psychol Bull. 2013:139(3):536–541. 23607431 10.1037/a0030515

[ref25] Gallagher HL, Happé F, Brunswick N, Fletcher PC, Frith U, Frith CD. Reading the mind in cartoons and stories: an fMRI study of “theory of mind” in verbal and nonverbal tasks. Neuropsychologia. 2000:38(1):11–21. 10617288 10.1016/s0028-3932(99)00053-6

[ref26] Gusnard DA, Akbudak E, Shulman GL, Raichle ME. Medial prefrontal cortex and self-referential mental activity: relation to a default mode of brain function. PNAS. 2001:98(7):4259–4264. 11259662 10.1073/pnas.071043098 PMC31213

[ref27] Ho NSP, Poerio G, Konu D, Turnbull A, Sormaz M, Leech R, Bernhardt B, Jefferies E, Smallwood J. Facing up to the wandering mind: patterns of off-task laboratory thought are associated with stronger neural recruitment of right fusiform cortex while processing facial stimuli. NeuroImage. 2020:214(October 2019):116765. 32213314 10.1016/j.neuroimage.2020.116765 PMC7284321

[ref28] Jöbsis F . Noninvasive, infrared monitoring of cerebral and myocardial oxygen sufficiency and circulatory parameters. Science. 1977:198(4323):1264–1267. 929199 10.1126/science.929199

[ref29] Johnson SC, Baxter LC, Wilder LS, Pipe JG, Heiserman JE, Prigatano GP. Neural correlates of self-reflection. Brain. 2002:125(8):1808–1814. 12135971 10.1093/brain/awf181

[ref30] Kam JWY, Dao E, Farley J, Fitzpatrick K, Smallwood J, Schooler JW, Handy TC. Slow fluctuations in attentional control of sensory cortex. J Cogn Neurosci. 2011:23(2):460–470. 20146593 10.1162/jocn.2010.21443

[ref31] Kaynezhad P, Mitra S, Bale G, Bauer C, Lingam I, Meehan C, Avdic-Belltheus A, Martinello KA, Bainbridge A, Robertson NJ, et al. Quantification of the severity of hypoxic-ischemic brain injury in a neonatal preclinical model using measurements of cytochrome-c-oxidase from a miniature broadband-near-infrared spectroscopy system. Neurophotonics. 2019:6(04):1. 10.1117/1.NPh.6.4.045009 PMC6855218 31737744

[ref32] Kelley WM, Macrae CN, Wyland CL, Caglar S, Inati S, Heatherton TF. Finding the self? An event-related fMRI study. J Cogn Neurosci. 2002:14(5):785–794. 12167262 10.1162/08989290260138672

[ref33] Konu D, Turnbull A, Karapanagiotidis T, Wang HT, Brown LR, Jefferies E, Smallwood J. A role for the ventromedial prefrontal cortex in self-generated episodic social cognition. NeuroImage. 2020:218(March):116977. 32450251 10.1016/j.neuroimage.2020.116977 PMC7422831

[ref34] Laureys S, Goldman S, Phillips C, Van Bogaert P, Aerts J, Luxen A, Franck G, Maquet P. Impaired effective cortical connectivity in vegetative state: preliminary investigation using PET. NeuroImage. 1999:9(4):377–382. 10191166 10.1006/nimg.1998.0414

[ref35] Lavie N . Perceptual load as a necessary condition for selective attention. J Exp Psychol Hum Percept Perform. 1995:21(3):451–468. 7790827 10.1037//0096-1523.21.3.451

[ref36] Lavie N . Distracted and confused?: selective attention under load. Trends Cogn Sci. 2005:9(2):75–82. 15668100 10.1016/j.tics.2004.12.004

[ref37] Lavie N, Beck DM, Konstantinou N. Blinded by the load: attention, awareness and the role of perceptual load. Philos Trans R Soc Lond B Biol Sci. 2014:369(1641):20130205. 24639578 10.1098/rstb.2013.0205 PMC3965161

[ref38] Lennie P . The cost of cortical computation. Curr Biol. 2003:13(6):493–497. 12646132 10.1016/s0960-9822(03)00135-0

[ref39] Logothetis NK . What we can do and what we cannot do with fMRI. Nature. 2008:453(7197):869–878. 18548064 10.1038/nature06976

[ref40] Mars RB, Neubert FX, Noonan MAP, Sallet J, Toni I, Rushworth MFS. On the relationship between the “default mode network” and the “social brain”. Front Hum Neurosci. 2012:6(JUNE 2012):1–9. 22737119 10.3389/fnhum.2012.00189 PMC3380415

[ref41] Mason MF, Norton MI, Van Horn JD, Wegner DM, Grafton ST, Macrae CN. Wandering minds: the default network and stimulus-independent thought. Science. 2007:315(5810):393–395. 17234951 10.1126/science.1131295 PMC1821121

[ref42] Mazoyer B, Zago L, Mellet E, Bricogne S, Etard O, Houdé O, Crivello F, Joliot M, Petit L, Tzourio-Mazoyer N. Cortical networks for working memory and executive functions sustain the conscious resting state in man. Brain Res Bull. 2001:54(3):287–298. 11287133 10.1016/s0361-9230(00)00437-8

[ref43] McKiernan KA, Kaufman JN, Kucera-Thompson J, Binder JR. A parametric manipulation of factors affecting task-induced deactivation in functional neuroimaging. J Cogn Neurosci. 2003:15(3):394–408. 12729491 10.1162/089892903321593117

[ref44] McKiernan KA, D'Angelo BR, Kaufman JN, Binder JR. Interrupting the “stream of consciousness”: an fMRI investigation. NeuroImage. 2006:29(4):1185–1191. 16269249 10.1016/j.neuroimage.2005.09.030 PMC1634934

[ref45] Mitra S, Kendall GS, Bainbridge A, Sokolska M, Dinan M, Uria-Avellanal C, Price D, Mckinnon K, Gunny R, Huertas-Ceballos A, et al. Proton magnetic resonance spectroscopy lactate/N-acetylaspartate within 2 weeks of birth accurately predicts 2-year motor, cognitive and language outcomes in neonatal encephalopathy after therapeutic hypothermia. Arch Dis Child Fetal Neonatal Ed. 2019:104(4):F424–F432. 30322975 10.1136/archdischild-2018-315478

[ref46] Molavi B, Dumont GA. Wavelet-based motion artifact removal for functional near-infrared spectroscopy. Physiol Meas. 2012:33(2):259–270. 22273765 10.1088/0967-3334/33/2/259

[ref47] Morris J, Keith Ngai MY, Yeomans MR, Forster S. A high perceptual load task reduces thoughts about chocolate, even while hungry. Appetite. 2020:151:104694. 32268163 10.1016/j.appet.2020.104694

[ref48] Ochsner KN, Knierim K, Ludlow DH, Hanelin J, Ramachandran T, Glover G, Mackey SC. Reflecting upon feelings: an fMRI study of neural systems supporting the attribution of emotion to self and other. J Cogn Neurosci. 2004:16(10):1746–1772. 15701226 10.1162/0898929042947829

[ref49] Ohta H, Yamada T, Watanabe H, Kanai C, Tanaka E, Ohno T, Takayama Y, Iwanami A, Kato N, Hashimoto R-i. An fMRI study of reduced perceptual load-dependent modulation of task-irrelevant activity in adults with autism spectrum conditions. NeuroImage. 2012:61(4):1176–1187. 22465842 10.1016/j.neuroimage.2012.03.042

[ref50] Patil AV, Safaie J, Moghaddam HA, Wallois F, Grebe R. Experimental investigation of NIRS spatial sensitivity. Biomed Opt Express. 2011:2(6):1478–1493. 21698012 10.1364/BOE.2.001478 PMC3114217

[ref51] Peeters-Scholte C, van den Tweel E, Groenendaal F, van Bel F. Redox state of near infrared spectroscopy-measured cytochrome aa3 correlates with delayed cerebral energy failure following perinatal hypoxia-ischaemia in the newborn pig. Exp Brain Res. 2004:156(1):20–26. 14689136 10.1007/s00221-003-1761-5

[ref52] Phan P, Highton D, Lai J, Smith M, Elwell C, Tachtsidis I. Multi-channel multi-distance broadband near-infrared spectroscopy system to measure the spatial response of cellular oxygen metabolism and tissue oxygenation. Biomed Opt Express. 2016:7(11):4424–4440. 27895985 10.1364/BOE.7.004424 PMC5119585

[ref53] Raichle ME, MacLeod AM, Snyder AZ, Powers WJ, Gusnard DA, Shulman GL. A default mode of brain function. Proc Natl Acad Sci. 2001:98(2):676–682. 11209064 10.1073/pnas.98.2.676 PMC14647

[ref54] Rees G, Frith CD, Lavie N. Modulating irrelevant motion perception by varying attentional load in an unrelated task. Science. 1997:278(5343):1616–1619. 9374459 10.1126/science.278.5343.1616

[ref55] Schooler JW, Smallwood J, Christoff K, Handy TC, Reichle ED, Sayette MA. Meta-awareness, perceptual decoupling and the wandering mind. Trends Cogn Sci. 2011:15(7):319–326. 21684189 10.1016/j.tics.2011.05.006

[ref56] Schwartz S, Vuilleumier P, Hutton C, Maravita A, Dolan RJ, Driver J. Attentional load and sensory competition in human vision: modulation of fMRI responses by load at fixation during task-irrelevant stimulation in the peripheral visual field. Cereb Cortex. 2005:15(6):770–786. 15459076 10.1093/cercor/bhh178

[ref57] Seli P, Ralph BCW, Konishi M, Smilek D, Schacter DL. What did you have in mind? Examining the content of intentional and unintentional types of mind wandering. Conscious Cogn. 2017:51(January):149–156. 28371688 10.1016/j.concog.2017.03.007 PMC5439521

[ref58] Singh KD, Fawcett IP. Transient and linearly graded deactivation of the human default-mode network by a visual detection task. NeuroImage. 2008:41(1):100–112. 18375149 10.1016/j.neuroimage.2008.01.051

[ref72] Smallwood J . Why the Global Availability of Mind Wandering Necessitates Resource Competition: Reply to McVay and Kane (2010). Psychol Bull. 2010:136(2):202–207.

[ref59] Smallwood J . Searching for the elements of thought: reply to Franklin, Mrazek, Broadway, and Schooler (2013). Psychol Bull. 2013:139(3):542–547. 23607432 10.1037/a0031019

[ref60] Smallwood J, Schooler JW. The science of mind wandering: empirically navigating the stream of consciousness. Annu Rev Psychol. 2015:66(1):487–518. 25293689 10.1146/annurev-psych-010814-015331

[ref61] Smallwood J, Brown K, Baird B, Schooler JW. Cooperation between the default mode network and the frontal–parietal network in the production of an internal train of thought. Brain Res. 2012:1428:60–70. 21466793 10.1016/j.brainres.2011.03.072

[ref62] Smallwood J, Bernhardt BC, Leech R, Bzdok D, Jefferies E, Margulies DS. The default mode network in cognition: a topographical perspective. Nat Rev Neurosci. 2021a:22(8):503–513. 34226715 10.1038/s41583-021-00474-4

[ref63] Smallwood J, Turnbull A, Wang H, Ho NSP, Poerio GL, Karapanagiotidis T, Konu D, Mckeown B, Zhang M, Murphy C, et al. The neural correlates of ongoing conscious thought. iScience. 2021b:24(3):1–15. 10.1016/j.isci.2021.102132 PMC7907463 33665553

[ref64] Sokoloff L, Mangold R, Wechsler RL, Kennedy C, Kety SS. The effect of mental arithmetic on cerebral circulation and metabolism. J Clin Invest. 1955:34(7 Pt 1):1101–1108. 14392225 10.1172/JCI103159 PMC438861

[ref65] Sormaz M, Murphy C, Wang HT, Hymers M, Karapanagiotidis T, Poerio G, Margulies DS, Jefferies E, Smallwood J. Default mode network can support the level of detail in experience during active task states. Proc Natl Acad Sci U S A. 2018:115(37):9318–9323. 30150393 10.1073/pnas.1721259115 PMC6140531

[ref66] Stawarczyk D, Majerus S, Maquet P, D'Argembeau A. Neural correlates of ongoing conscious experience: both task-unrelatedness and stimulus-independence are related to default network activity. PLoS One. 2011:6(2):e16997. 21347270 10.1371/journal.pone.0016997 PMC3038939

[ref67] Svoboda E, McKinnon MC, Levine B. The functional neuroanatomy of autobiographical memory: a meta-analysis. Neuropsychologia. 2006:44(12):2189–2208. 16806314 10.1016/j.neuropsychologia.2006.05.023 PMC1995661

[ref68] Torralbo A, Kelley TA, Rees G, Lavie N. Attention induced neural response trade-off in retinotopic cortex under load. Sci Rep. 2016:6(September):1–10. 27625311 10.1038/srep33041 PMC5021995

[ref69] Turnbull A, Wang HT, Murphy C, Ho NSP, Wang X, Sormaz M, Karapanagiotidis T, Leech RM, Bernhardt B, Margulies DS, et al. Left dorsolateral prefrontal cortex supports context-dependent prioritisation of off-task thought. Nat Commun. 2019:10(1):3816. 31444333 10.1038/s41467-019-11764-y PMC6707151

[ref70] Ye JC, Tak S, Jang KE, Jung J, Jang J. NIRS-SPM: statistical parametric mapping for near-infrared spectroscopy. NeuroImage. 2009:44(2):428–447. 18848897 10.1016/j.neuroimage.2008.08.036

[ref71] Zhang M, Bernhardt BC, Wang X, Varga D, Krieger-Redwood K, Royer J, Rodríguez-Cruces R, Vos de Wael R, Margulies DS, Smallwood J, et al. Perceptual coupling and decoupling of the default mode network during mind-wandering and reading. elife. 2022:11:e74011. 10.7554/eLife.74011 PMC8937216 35311643

