# Supplementary material for: On the coupling and decoupling of mind wandering and perception: a shared metabolism account
Source: Cereb Cortex Commun. 2023 Nov 21;4(4):tgad021. doi: 10.1093/texcom/tgad021 (PMC10703546; doi:10.1093/texcom/tgad021)
Supplement: SupplementaryMaterials_tgad021 [file supplementarymaterials_tgad021.docx]

**Supplementary Materials**

**Supplementary Table 1**

*Average Channel Positions and Brodmann Area Allocations (in bold)*

| Channels | MNI Coordinates | | | Brodmann  Areas | Probability |
| --- | --- | --- | --- | --- | --- |
|  | X | Y | Z |  |  |
| 1 | 53.76 | 23.69 | 37.51 |  |  |
|  |  |  |  | 9 | 0.08 |
|  |  |  |  | **44** | 0.73 |
|  |  |  |  | 45 | 0.19 |
|  |  |  |  |  |  |
| 2 | 42.35 | 35.90 | 42.98 |  |  |
|  |  |  |  | **9** | 0.66 |
|  |  |  |  | 44 | 0.03 |
|  |  |  |  | 45 | 0.12 |
|  |  |  |  | 46 | 0.19 |
|  |  |  |  |  |  |
| 3 | 56.37 | 34.04 | 20.31 |  |  |
|  |  |  |  | **45** | 1.00 |
|  |  |  |  |  |  |
| 4 | 44.92 | 48.25 | 26.69 |  |  |
|  |  |  |  | 45 | 0.47 |
|  |  |  |  | **46** | 0.53 |
|  |  |  |  |  |  |
| 5 | 24.71 | 46.08 | 47.20 |  |  |
|  |  |  |  | **9** | 1.00 |
|  |  |  |  |  |  |
| 6 | 10.22 | 51.27 | 48.24 |  |  |
|  |  |  |  | 8 | 0.02 |
|  |  |  |  | **9** | 0.98 |
|  |  |  |  |  |  |
| 7 | 27.84 | 58.27 | 31.43 |  |  |
|  |  |  |  | 9 | 0.25 |
|  |  |  |  | 10 | 0.17 |
|  |  |  |  | **46** | 0.58 |
|  |  |  |  |  |  |
| 8 | 11.08 | 63.82 | 32.80 |  |  |
|  |  |  |  | 9 | 0.35 |
|  |  |  |  | **10** | 0.65 |
|  |  |  |  |  |  |
| 9 | -10.16 | 52.61 | 46.57 |  |  |
|  |  |  |  | **9** | 1.00 |
|  |  |  |  |  |  |
| 10 | -24.96 | 47.47 | 43.22 |  |  |
|  |  |  |  | **9** | 0.94 |
|  |  |  |  | 46 | 0.06 |
|  |  |  |  |  |  |
| 11 | -10.16 | 64.33 | 30.94 |  |  |
|  |  |  |  | 9 | 0.25 |
|  |  |  |  | **10** | 0.75 |
|  |  |  |  |  |  |
| 12 | -29.35 | 58.49 | 26.88 |  |  |
|  |  |  |  | 10 | 0.17 |
|  |  |  |  | **46** | 0.83 |
|  |  |  |  |  |  |
| 13 | -42.49 | 35.96 | 39.18 |  |  |
|  |  |  |  | **9** | 0.36 |
|  |  |  |  | 44 | 0.06 |
|  |  |  |  | 45 | 0.30 |
|  |  |  |  | 46 | 0.27 |
|  |  |  |  |  |  |
| 14 | -55.35 | 20.45 | 30.96 |  |  |
|  |  |  |  | **44** | 0.77 |
|  |  |  |  | 45 | 0.23 |
|  |  |  |  |  |  |
| 15 | -45.57 | 46.57 | 22.59 |  |  |
|  |  |  |  | **45** | 0.62 |
|  |  |  |  | 46 | 0.38 |
|  |  |  |  |  |  |
| 16 | -56.55 | 29.08 | 16.12 |  |  |
|  |  |  |  | 44 | 0.07 |
|  |  |  |  | **45** | 0.93 |

Overview of group-averaged MNI coordinates and assignment to Brodmann Areas.
